# Breast density and impacts on health

**DOI:** 10.3332/ecancer.2017.ed70

**Published:** 2017-08-08

**Authors:** Cheryl Cruwys, JoAnn Pushkin

**Affiliations:** 1 Breast Density Matters UK, Masmoulineix, 87380, Glanges, France; 2 DenseBreast-info.org, PO Box 997, Deer Park, NY 11729, USA

**Keywords:** breast cancer, breast density, dense breasts, mammography, ultrasound, early detection

## Abstract

The World Health Organization states ‘Early detection in order to improve breast cancer outcome and survival remains the cornerstone of breast cancer control’ [WHO (World Health Organization) (2017) **Breast cancer: prevention and control** Available from: http://www.who.int/cancer/detection/breastcancer/en/]. Breast Density Matters UK is a non-profit breast cancer organization. The organization’s mission is to educate about breast density and its screening and risk implications with the goal of achieving the earliest stage diagnosis possible for women with dense breasts. This educational mission is endorsed by breast imaging experts worldwide [Berg WA (2015–2017) **Dense Breast-info Inc** Available from: http://densebreast-info.org/about.aspx].

## Introduction

Breast density has implications for both breast screening and risk [3]. Dense breast tissue both obscures cancers on a mammogram and is also an independent risk factor for the development of breast cancer. Whilst dense breasts are common and not abnormal, it is known that mammograms are less effective in dense breasts and supplemental screening can increase the detection of early stage breast cancer in dense breasts. Cheryl Cruwys, co-founder of Breast Density Matters UK, was diagnosed with breast cancer in May 2016. Her breast screening was conducted in France and because she has dense breast tissue, she received a supplemental ultrasound. The ultrasound detected what the mammogram did not, an asymptomatic 8mm invasive tumour. Found early, her treatment was minimal with a positive health outcome. Incidences have been reported of women, with dense breasts, who have been diagnosed with later stage, more advanced cancers whilst having previously received ‘normal’ mammograms [4]. Breast Density Matters UK supports the dissemination of personal density information to women so that they can be informed participants in their own breast health surveillance.

Each year in the UK, approximately 3,500 breast cancers are missed by mammography [5] and, each year, approximately 11,000 people die from breast cancer in the UK [6]. Cancers missed by mammography, known as ‘interval cancers’ (cancers detected because of symptoms, such as a lump, during the interval between a ‘normal’ screening and next screening), are then detected when later stage, more aggressive and with worse outcomes [7].

A study found women with the densest breasts, categorized as ‘extremely dense’ (>75% density), were 17 times more likely to have an interval cancer than women with fatty breasts [8].

Breast Density Matters UK believes that supplemental screening in dense breasts, after mammography, has the potential to save lives by reducing the incidence of breast cancer diagnosed at later stage, when less treatable and survivable.

## Clinical evidence

A growing pool of clinical research provides substantial evidence that supplemental screening detects more cancers than by mammography alone. At the 2016 European Breast Cancer Conference in Amsterdam, findings from the ASTOUND Study [9] were presented. Professor Emiel Rutgers, of the Netherlands Cancer Institute stated, ‘the advances could offer help to a large number of women who were not served well by the current system of mammograms. Women with dense breasts face a real problem. First they have an increased risk for developing breast cancer… secondly the mammogram is not helpful enough, small cancers are obscured by dense breast tissue’ [5].

A recently published large scale study on breast cancer risk, which involved over 200,000 women, found that breast density is the most prevalent of all common risk factors including personal and family history of the disease [10]. Table 1 details recent clinical studies on the topics of supplemental screening, diagnosis and treatment costs.

## Screening in dense breasts around the world

In France, in women with extremely dense breasts, mammograms and supplemental ultrasound have been coupled for many years [14]. The Austrian Breast Cancer Early Detection Programme also provides for ultrasound screening after mammography if breast tissue is found to be dense. In the USA, 31 states have passed legislation to inform women about breast density following a mammogram and supplemental screening is generally extended if ordered by a physician. In Australia, a group of scientists have recently formed an advocacy [15] effort encouraging the density discussion and have held public forums to educate on breast density.

Since 2016, Breast Density Matters UK has attended several breast cancer conferences/symposiums in order to coalition build around the mission of education about breast density. As outreach and activity are determined by funding, we work to secure educational and public engagement funding to continue our mission of encouraging informed conversations about breast density in the UK and worldwide.

## Conclusion

Breast Density Matters UK aims to educate about breast density, liaising worldwide with breast imaging researchers, medical experts and advocates with the following mission:

- Educate about screening and risk implications of dense tissue.
- Encourage informed decisions for women; early diagnosis; positive health outcomes

For more information about breast density or the work of Breast Density Matters UK, please watch this video: http://ecancer.org/video/5887/identifying-cancer-in-dense-breast-tissue.php [16] and feel free to contact us.

**Email**: breastdensitymatters.uk@gmail.com

**Facebook**: Breast Density Matters UK

**Twitter**: @Cheryl_Cruwys

## Figures and Tables

**Table 1.** Recent clinical studies on the topics of supplemental screening, diagnosis and treatment cost include.

Topic/Study	Conclusion
*Supplemental Screening*	
Japan Strategic Anti-cancer Randomized Trial (J-START) Sensitivity and specificity of mammography and adjunctive ultrasonography to screen for breast cancer	Adjunctive ultrasonography was associated with a significantly higher detection rate of breast cancer than mammography alone. Thus, ultrasonography could offer a low-cost way to increase sensitivity and detection rates of early cancers in women with dense tissue [11].
*Early Diagnosis*	
Influence of tumour stage at breast cancer detection on survival in modern times: population based study in 173,797 patients	The diagnosis of breast cancer at an early stage remains vital [12].
*Cost Implications of Late Stage Diagnosis*	
Comparison of Treatment Costs for Breast Cancer, by Tumor Stage and Type of Service	Treating advanced versus early-stage breast cancer is associated with significant increases in incremental costs. Knowledge of the stage-specific cost data provides support for strengthening programs, such as breast cancer screening, that are designed to shift breast cancer diagnosis to earlier disease stages [13].
